# Virus-like nanoparticle vaccines for inducing long-lasting immunity against infectious diseases

**DOI:** 10.1093/nsr/nwae032

**Published:** 2024-01-23

**Authors:** Yi-Nan Zhang, Sarah Auclair, Jiang Zhu

**Affiliations:** Department of Integrative Structural and Computational Biology, The Scripps Research Institute, USA; Department of Integrative Structural and Computational Biology, The Scripps Research Institute, USA; Department of Integrative Structural and Computational Biology, The Scripps Research Institute, USA; Department of Immunology and Microbiology, The Scripps Research Institute, USA

Prophylactic vaccines that can generate long-lasting protective immune responses are needed for many infectious diseases. Efficient vaccination requires vaccine antigens to be retained and presented to B cells in lymph node follicles to induce robust and long-lived germinal center (GC) reactions [1,2]. The delivery of viral antigens in a multimeric form using nanoparticle carriers to mimic virus-like particles has become a trend in recent vaccine research [3,4]. With virus-like structural features (size, shape, and a dense display of viral antigens), nanoparticles travel through lymphatics and are retained in lymph nodes [5]. These nanoparticles can serve as delivery systems by integrating the antigen display and adjuvant conjugation for more effective cellular interactions and stimulate more potent and long-lasting immune responses than soluble antigens [6,7]. However, the engineering of nanoparticles with multifunctional components remains a challenge.

**Figure 1.**
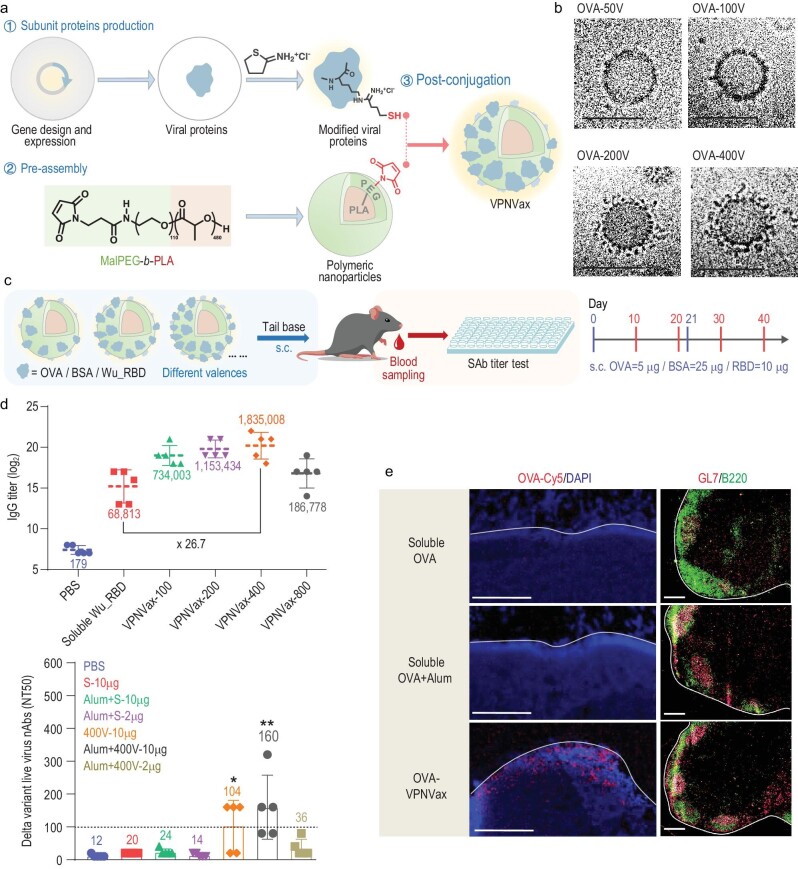
(a) Schematic illustration of VPNVax preparation. (b) Cryo-EM images of OVA-VPNVaxs with variable antigen valencies (scale bar = 50 nm). (c) Schematic representation of the mouse immunization regimen for VPNVaxs with different antigens and valencies. (d) Top: VPNVax-induced binding antibody responses against Wu_RBD. Bottom: VPNVax-induced neutralizing antibody responses against live SARS-CoV-2 virus (Delta variant). (e) OVA-VPNVax accumulation and vaccine-induced germinal centers in lymph node follicles at 2 weeks after a single-dose injection (scale bar = 2 μm). Reprinted with permission from ref. [8].

Recently, a team led by professors Xuesi Chen, Wantong Song, and MingYao Tian at the Changchun Institute of Applied Chemistry, Chinese Academy of Sciences and Changchun Veterinary Research Institute, Chinese Academy of Agricultural Sciences published a work in *National Science Review* (Fig. 1) [8] reporting a multifunctional viromimetic polymer nanoparticle vaccine (VPNVax) against SARS-CoV-2, in which the receptor-binding domain (RBD) proteins were conjugated to the surface of a polymeric nanoparticle with TLR7/8 agonists encapsulated inside for self-adjuvantation.

In this study, self-assembling polymeric polyethylene glycol-*b*-polylactic acid (PEG-*b*-PLA) was selected as a nanoparticle platform (Fig. 1a) [8]. Subunit proteins of vaccine antigens were conjugated onto the surface of a polymeric nanoparticle through a click chemical reaction to generate a dual-component VPNVax. This system allows the rapid replacement of viral antigens to match emerging viruses. Ovalbumin (OVA) was first used as a model antigen to study the physicochemical properties (size and antigen valency) of VPNVaxs. The morphology and structural integrity of OVA-VPNVaxs were validated by cryogenic transmission electron microscopy (cryo-TEM). EM images demonstrated well-formed particles with a tunable antigen valency for all four VPNVax samples (Fig. 1b).

VPNVaxs were then constructed with different antigen valencies to test their immunogenicity in a mouse model (Fig. 1c) [8]. The serological analysis revealed that the SARS-CoV-2 vaccine-induced antibody responses were influenced by the antigen form (soluble antigen vs. multivalent presentation of antigens on a nanoparticle), antigen valency on the surface of nanoparticles, vaccine dose, and adjuvant properties (Fig. 1d). Overall, adjuvanted VPNVax with optimal antigen valency elicited the highest binding antibody and neutralizing antibody titers, indicating the importance of antigen design and display on nanoparticle platforms. The mechanism of VPNVax-induced immunity was also investigated. Compared with the soluble antigen, the VPNVax showed significantly longer retention (over two weeks) in draining lymph node follicles and stronger GC reactions (Fig. 1e). Notably, the methods used to track nanoparticle antigens in lymph nodes may result in different experimental outcomes. This study conjugated fluorescent dyes to the model antigen OVA, which may have affected the readout due to their fluorescent signal decay over time. An alternative approach to studying the trafficking and retention of nanoparticle vaccines is to quantify the signal through immunostaining of the viral antigens using antigen-specific binding/neutralizing antibodies [9,10].

In summary, this study demonstrated a novel dual-component nanoparticle vaccine platform that consists of a self-assembling polymeric core and surface conjugated antigens [8]. This VPNVax platform has the potential to incorporate adjuvants, such as toll-like receptor agonists, to further regulate the innate and adaptive immune responses. The understanding obtained from this study will facilitate the rational design and development of more effective polymer-based virus-like particle vaccines.
